# Supplementary material for: Understanding Reasons for Vaccination Hesitancy and Implementing Effective Countermeasures: An Online Survey of Individuals Unvaccinated against COVID-19
Source: Vaccines (Basel). 2024 May 5;12(5):499. doi: 10.3390/vaccines12050499 (PMC11125705; doi:10.3390/vaccines12050499)
Supplement: Supplementary file 1 [file vaccines-12-00499-s001.zip › vaccines-2919943-supplementary.pdf]

Table S1. Structure of the questionnaire.

Questions	Options	Answer type
Sex		single-answer
	Male	
	Female	
Age		number-completion
Marriage		single-answer
	Not married (including divorced or bereaved)	
	married	
Have children		single-answer
	Yes	
	No	
Family income		single-answer
	Less than 2 million yen	
	2–4 million yen	
	4–6 million yen	
	6–8 million yen	
	8–10 million yen	
	10–12 million yen	
	12–15 million yen	
	15–20 million yen	
	More than 20 million yen	
	Do not know	
Private income		single-answer
	Less than 2 million yen	
	2–4 million yen	
	4–6 million yen	
	6–8 million yen	
	8–10 million yen	
	10–12 million yen	
	12–15 million yen	
	15–20 million yen	
	More than 20 million yen	
	Do not know	
Occupation		single-answer
	Unemployed	
	Part-time job	
	Housewife (househusband)	
	Company employee (other)	
	Company employee (clerical)	
	Self-employed	
	Company employee (technical)	
	Free Business	
	Student	
	Other (free text)	
	Civil servants	
	Manager/Officer	
Student category		single-answer
	Vocational school student	

	Junior college student	
	College student	
	Graduate student	
	Others	
Number of COVID-19 vaccination		single-answer
	0	
	1	
	2	
	3	
	4	
	more than 5	
Reasons for declining COVID-19 vaccination		
Busy.	Likert scale	single-answer
Convenience to the venue is not good.	Likert scale	single-answer
Booking is troublesome.	Likert scale	single-answer
COVID-19 infection does not cause severe illness.	Likert scale	single-answer
No infection with COVID-19.	Likert scale	single-answer
Vaccine ineffectiveness.	Likert scale	single-answer
Already infected with COVID-19.	Likert scale	single-answer
Other infection control measures.	Likert scale	single-answer
Concerns about adverse reactions.	Likert scale	single-answer
Concerns about vaccine safety.	Likert scale	single-answer
Concerns about long-term effects of the vaccine.	Likert scale	single-answer
Pregnant and worried about the impact on the fetus.	Likert scale	single-answer
Concerns about impact on pre-existing medical conditions.	Likert scale	single-answer
There is distrust of vaccine development and regulators.	Likert scale	single-answer
Information from the government cannot be trusted.	Likert scale	single-answer
Distrust of healthcare in general.	Likert scale	single-answer
Few people around me have been infected with the COVID-19.	Likert scale	single-answer
Few people around me are vaccinated.	Likert scale	single-answer
People around me experienced severe adverse reactions.	Likert scale	single-answer
My doctor told me.	Likert scale	single-answer
Religious reason.	Likert scale	single-answer
Comorbidity		multiple-answer
	Hypertension	
	Diabetes mellitus	
	Bronchial asthma	
	Anaphylactic shock	
	Psychiatric disorders	
	Gout	
	Lipid disorders	
	Rheumatoid arthritis	
	Respiratory diseases	
	Cardiovascular diseases	

	Allergic diseases	
	Immunodeficiency diseases	
	Malignant tumors	
	Thyroid diseases	
	Liver disease	
	Kidney disease	
	Others	free-text answer
	None	
Influenza vaccination after 2019.		single-answer
	Yes	
	None	
Infected with SARS-CoV-2		single-answer
	Yes	
	No	
Symptoms after SARS-CoV-2 infection.		multiple-answer
	No symptoms	
	fever	
	Cough	
	Sore throat	
	Headache	
	Joint and muscle pain	
	Taste or smell disorders	
	diarrhea	
	Lethargy	
	Breathlessness	
	Inability to speak, move or consciousness disorders	
	Skin rashes	
	Eye irritation or pain	
	Chest pain	
	Other	free-text answer
COVID-19 conspiracy beliefs		
Big pharma is encouraging the spread of COVID-19 to make money		single-answer
	Yes	
	No	
COVID-19 was developed by the government as part of a bioweapons program.		single-answer
	Yes	
	No	
5G is causing the COVID-19		single-answer
	Yes	
	No	
The COVID-19 is a myth to force vaccinations on people		single-answer
	Yes	
	No	
There is no such thing as the COVID-19		single-answer
	Yes	

	No	
Trusted sources		
Facebook	Likert scale	single-answer
Twitter	Likert scale	single-answer
Instagram	Likert scale	single-answer
YouTube	Likert scale	single-answer
Government	Likert scale	single-answer
Public health center	Likert scale	single-answer
Television	Likert scale	single-answer
Newspaper	Likert scale	single-answer
Weekly magazine	Likert scale	single-answer
Primary care physician	Likert scale	single-answer
Healthcare professional	Likert scale	single-answer
Pharmaceutical company	Likert scale	single-answer
Friend	Likert scale	single-answer
Family	Likert scale	single-answer
Others (free text)	Likert scale	single-answer
What information do you want to know about COVID-19 vaccine?		multiple-answer
	Adverse Reactions	
	Safety	
	Efficacy	
	Genetic Effects	
	Cost	
	Future COVID-19 vaccination schedule	
	None in particular	
Disadvantages of not vaccinated		multiple-answer
	Discriminated against for not vaccinating	
	Forced to vaccinate	
	I was fired from my job	
	Infected with SARS-CoV-2	
	Other	free-text answer
	None in particular	

The Likert scale options were as follows: 1, not at all applicable; 2, not very much; 3, cannot say either way; 4, somewhat true; and 5, very applicable.

Table S2. Tabulation of reasons for declining COVID-19 vaccination

	Very applicable	Somewhat true	Cannot say either way	Not very much	Not at all applicable
Busy.	20 (7.6)	30 (11.5)	77 (29.4)	33 (12.6)	102 (38.9)
Convenience to the venue is not good.	21 (8.0)	22 (8.4)	86 (32.8)	39 (14.9)	94 (35.9)
Booking is troublesome.	43 (16.4)	41 (15.6)	67 (25.6)	34 (13)	77 (29.4)
COVID-19 infection does not cause severe illness.	26 (9.9)	42 (16)	101 (38.5)	33 (12.6)	60 (22.9)
No infection with COVID-19.	14 (5.3)	15 (5.7)	107 (40.8)	33 (12.6)	93 (35.5)
Vaccine ineffectiveness.	58 (22.1)	33 (12.6)	104 (39.7)	34 (13)	33 (12.6)
Already infected with COVID-19.	37 (14.1)	17 (6.5)	39 (14.9)	13 (5)	156 (59.5)
Other infection control measures.	34 (13)	51 (19.5)	74 (28.2)	26 (9.9)	77 (29.4)
Concerns about adverse reactions.	154 (58.8)	56 (21.4)	29 (11.1)	6 (2.3)	17 (6.5)
Concerns about vaccine safety.	147 (56.1)	52 (19.8)	35 (13.4)	10 (3.8)	18 (6.9)
Concerns about long-term effects of the vaccine.	107 (40.8)	53 (20.2)	59 (22.5)	16 (6.1)	27 (10.3)
Pregnant and worried about the impact on the fetus.	7 (2.7)	8 (3.1)	41 (15.6)	10 (3.8)	196 (74.8)
Concerns about the impact on pre-existing medical conditions.	34 (13.0)	34 (13.0)	59 (22.5)	38 (14.5)	97 (37.0)
There is distrust of vaccine development and regulators.	95 (36.3)	58 (22.1)	55 (21.0)	18 (6.9)	36 (13.7)
Information from the government cannot be trusted.	88 (33.6)	58 (22.1)	65 (24.8)	20 (7.6)	31 (11.8)
Distrust of healthcare in general.	41 (15.6)	57 (21.8)	81 (30.9)	35 (13.4)	48 (18.3)
Few people around me have been infected with the COVID-19.	32 (12.2)	56 (21.4)	69 (26.3)	53 (20.2)	52 (19.8)
Few people around me are vaccinated.	15 (5.7)	20 (7.6)	83 (31.7)	61 (23.3)	83 (31.7)
People around me experienced severe adverse reactions.	40 (15.3)	47 (17.9)	59 (22.5)	35 (13.4)	81 (30.9)
My doctor told me.	8 (3.1)	8 (3.1)	48 (18.3)	20 (7.6)	178 (67.9)
Religious reason.	3 (1.1)	1 (0.4)	34 (13)	19 (7.3)	205 (78.2)

Table S3. Tabulation of media that the participants trust

	Very applicable	Somewhat true	Cannot say either way	Not very much	Not at all applicable
Facebook	4 (1.5)	13 (5.0)	74 (28.2)	32 (12.2)	139 (53.1)
Twitter	16 (6.1)	38 (14.5)	73 (27.9)	37 (14.1)	98 (37.4)
Instagram	8 (3.1)	17 (6.5)	82 (31.3)	34 (13.0)	121 (46.2)
YouTube	21 (8.0)	37 (14.1)	85 (32.4)	30 (11.5)	89 (34.0)
Government	10 (3.8)	32 (12.2)	91 (34.7)	36 (13.7)	93 (35.5)
Public health center	8 (3.1)	46 (17.6)	94 (35.9)	30 (11.5)	84 (32.1)
Television	22 (8.4)	43 (16.4)	70 (26.7)	40 (15.3)	87 (33.2)
Newspaper	12 (4.6)	28 (10.7)	80 (30.5)	44 (16.8)	98 (37.4)
Weekly magazine	2 (0.8)	14 (5.3)	77 (29.4)	48 (18.3)	121 (46.2)
Primary care physician	9 (3.4)	48 (18.3)	94 (35.9)	28 (10.7)	83 (31.7)
Healthcare professional	11 (4.2)	51 (19.5)	94 (35.9)	23 (8.8)	83 (31.7)
Pharmaceutical company	3 (1.1)	21 (8.0)	95 (36.3)	39 (14.9)	104 (39.7)
Friend	15 (5.7)	43 (16.4)	108 (41.2)	30 (11.5)	66 (25.2)
Family	23 (8.8)	51 (19.5)	93 (35.5)	31 (11.8)	64 (24.4)
Others	21 (8.0)	6 (2.3)	46 (17.6)	5 (1.9)	184 (70.2)

Table S4 Basic characteristics of participants by clusters

Cluster (N)<br>Variable (n (%))	1 (28)	2 (85)	3 (149)	Univariable p-value
Female	6 (21.4)	50 (58.8)	70 (47.0)	**0.002**
Age (mean [SD])	42.5 [13.9]	50.5 [13.5]	47.6 [15.9]	**0.046**
Marriage	11 (39.3)	40 (47.1)	57 (38.3)	0.41
Have Children	10 (35.7)	47 (55.3)	61 (40.9)	**0.012**
Family income				**0.039**
Less than 2 million yen	1 (3.6)	9 (10.6)	29 (19.5)	
2–4 million yen	5 (17.9)	25 (29.4)	31 (20.8)	
4–6 million yen	7 (25.0)	15 (17.6)	31 (20.8)	
6–8 million yen	4 (14.3)	6 (7.1)	15 (10.1)	
8–10 million yen	3 (10.7)	6 (7.1)	9 (6.0)	
10–12 million yen	0 (0.0)	4 (4.7)	2 (1.3)	
12–15 million yen	1 (3.6)	2 (2.4)	2 (1.3)	
15–20 million yen	0 (0.0)	0 (0.0)	1 (0.7)	
More than 20 million yen	0 (0.0)	1 (1.2)	0 (0.0)	
Do not know	6 (21.4)	17 (20.0)	29 (19.5)	
Private income				**0.034**
Less than 2 million yen	8 (28.6)	35 (41.2)	69 (46.3)	
2–4 million yen	4 (14.3)	23 (27.1)	33 (22.1)	
4–6 million yen	8 (28.6)	7 (8.2)	14 (9.4)	
6–8 million yen	1 (3.6)	2 (2.4)	5 (3.4)	
8–10 million yen	0 (0.0)	3 (3.5)	5 (3.4)	
10–12 million yen	0 (0.0)	0 (0.0)	1 (0.7)	
12–15 million yen	0 (0.0)	0 (0.0)	0 (0.0)	
15–20 million yen	0 (0.0)	1 (1.2)	0 (0.0)	
More than 20 million yen	6 (21.4)	14 (16.5)	21 (14.1)	
Do not know				
Occupation				0.051
Civil servants	0 (0.0)	1 (1.2)	2 (1.3)	
Manager/Officer	0 (0.0)	2 (2.4)	1 (0.7)	
Company employee (clerical)	3 (10.7)	9 (10.6)	10 (6.7)	
Company employee (technical)	5 (17.9)	2 (2.4)	12 (8.1)	
Company employee (other)	2 (7.1)	12 (14.1)	22 (14.8)	
Self-employed	2 (7.1)	9 (10.6)	11 (7.4)	
Freelancer	2 (7.1)	2 (2.4)	3 (2.0)	
Housewife (househusband)	3 (10.7)	11 (12.9)	25 (16.8)	
Part-time job	5 (17.9)	14 (16.5)	23 (15.4)	
Student	0 (0.0)	0 (0.0)	6 (4.0)	
Other	0 (0.0)	3 (3.5)	2 (1.3)	
Unemployed	6 (21.4)	20 (23.5)	32 (21.5)	
Comorbidity	4 (14.3)	27 (31.8)	53 (35.6)	**0.011**
Influenza vaccination after 2019.	1 (0.4)	8 (9.4)	12 (8.1)	**0.002**
Infected with SARS-CoV-2	6 (21.4)	25 (29.4)	36 (24.2)	**0.002**
Conspiracy theory	5 (17.9)	27 (31.8)	56 (37.6)	**0.010**
Experience Disadvantages of not vaccinated	2 (7.1)	16 (18.8)	20 (13.4)	**0.006**

We conducted univariable multinominal logistic regression analysis. The significant values are indicated in bold.

According to the exchange rate, as of August 2, 2023, 1 Japanese yen was equal to 0.0070 US dollars.
